# Digital science platform: an interactive web application and database of osteological material for anatomy education

**DOI:** 10.1186/s12909-022-03408-5

**Published:** 2022-05-12

**Authors:** Piotr Regulski, Jacek Tomczyk, Mariusz Białowarczuk, Wojciech Nowak, Marek Niezgódka

**Affiliations:** 1 Center of Digital Science and Technology, Cardinal Stefan Wyszynski University in Warsaw, Warsaw, Poland; 2 Department of Dental and Maxillofacial Radiology, Faculty of Medicine and Dentistry, Medical University of Warsaw, Binieckiego 6, 02-097 Warsaw, Poland; 3 Institute of Biological Sciences, Cardinal Stefan Wyszynski University in Warsaw, Warsaw, Poland

**Keywords:** Anatomy, Visualization, Education, Digital Science Platform, Pandemic, COVID-19

## Abstract

**Background:**

To meet the remote-learning constraints imposed due to the COVID-19 pandemic, the Digital Science Platform was developed. Human anatomy courses require practical classes that involve working on prepared specimens, although access to such specimens has been restricted. Therefore, the aim was to prepare appropriate-quality, scanned 3D model databases of human bone specimens and an interactive web application for universal access to educational materials.

**Main body:**

The database is located on the pcn.cnt.edu.pl website and contains 412 three-dimensional osteological models created via a structured light scanner, tomography and microtomography. The webservice contains a search engine and enables interactive visualization of the models. The database can be accessed, without restrictions, by any student or researcher wishing to use the models for noncommercial purposes. The stored models can be visualized with the open-source VisNow platform, which is also available to download from the webservice. The MariaDB backend database was deployed, and an Apache server with a personal home page (PHP) frontend was used.

**Conclusion:**

The models in the database are unique due to the specific digitalization process and skeleton specimen origin. Further development of the Digital Science Platform is foreseen in the near future to digitize other valuable materials.

## Background

In the face of the new COVID-19 pandemic teaching and learning conditions, it has become urgent to prepare widely accessible educational materials of adequate quality for universal access [1]. Study programmes for many fields (especially medical and biological programmes) include human anatomy courses. Practical classes require students to work on prepared specimens, which are not always available in teaching centres. This state of affairs is exacerbated by the extreme conditions requiring remote learning during the pandemic [2].

The available literature repeatedly emphasizes the need to move from a 2D representation of anatomical structures in atlases and textbooks to computer-aided 3D visualization [3, 4]. Anatomy education requires an understanding of the position and size of structures in space and involves the identification of landmarks in specimens and clinical images [5]. Three-dimensional digital anatomical models show potential in improving anatomy learning ability and cognition, particularly in students with low spatial visualization skills [6]. Interactive 3D models have a great impact by supporting students with a unique opportunity to employ the models in the training and study process [7]. The incorporation of digital technologies provides students with tools to increase their knowledge and better transition from basic to clinical studies [7, 8].

Therefore, the concept of preparing appropriate-quality 3D model databases for universal access to such educational materials was developed. The models represent specimens with a 1:1 scale that were previously available only in lecture halls and can be used by students wishing to learn or expand their knowledge of anatomy.

The presented database includes osteological specimens that can help students master the structure of the human skeleton. The motivation for the development of these specimens is related to the fact that we are currently dealing with an insufficient number of these prepared specimens, the classes during which students have access to them are rather short in duration, and the specimens are rather fragile [9]; this means that students are often tested on bone structures that they have never seen.

The high quality of these models can make them a unique tool for research teams in the preparation of anatomical materials for publication. These models can also be printed on almost all commercially available 3D printers.

The aim was to develop an interactive web application and database containing a dataset of scanned three-dimensional osteological models.

### Construction and content

The webservice and database are located on the pcn.cnt.edu.pl website [10] and contain the scanned 3D models of skeletons representing a 1:1 scale compared to real specimens. The specimens originated from a male skeleton aged 35/39 years old. The assessment of skeletal age and sex was made on the basis of skeletal determinants (including skull and pelvis features) used in anthropology [11, 12]. The skeleton came from Radom, from the municipal cemetery that operated during the years 1794–1811.

**Fig. 1:**
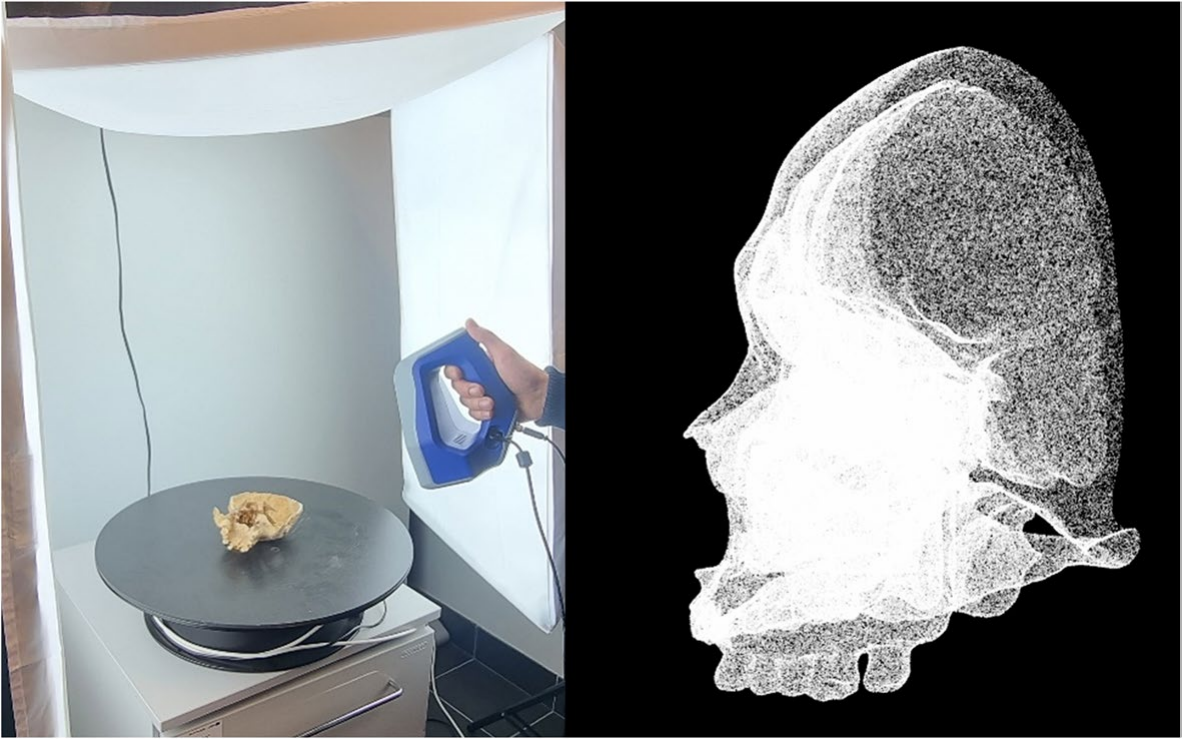
Process of scanning a handheld structured light scanner. The scan data were obtained as a point cloud from which the model was created. This figure was prepared by the authors

Several 3D scanning methods were used in the process of digitizing the bone material to obtain the most accurate representation of the surface. Most of the bone models were developed using a handheld structured light scanner [13]. The initial scan data (point cloud) were triangulated, and the obtained 3D models were characterized by their high accuracy and file weight (Fig. 1). The ability to view these data using a web browser (including a mobile version) required a significant reduction in the number of triangles in the model while retaining as much information as possible. In the processes of so-called “retopology”, the number of triangles in the models was reduced, and UV maps were prepared [14]. UV mapping is a process involving the projection of a 2D image onto the surface of a 3D model for texture mapping. Textures imitating bone material were also prepared. In cases where the object surfaces were inaccessible to the scanner, tomography was additionally utilized. The results of such tomography were then postproductionally combined with those obtained using the structured light scanner.

A total of 412 bone models are accessible from the webservice. Two hundred six of these are simplified models that can be directly accessed via a web browser with the embedded SketchFab Viewer application programming interface (API) and are available for download directly from the pcn.cnt.edu.pl website. Access to the models in the database is available through the implemented search engine or based on hierarchical categories (location of the bone) (Table 1).

**Table 1:** Listing of the osteological specimen with URLs to specific sections of the database

Section	Models	URL
Cranium	Frontal bone, Parietal bone, Occipital bone, Sphenoid bone, Temporal bone, Auditory ossicles, Maxilla, Mandible, Hyoid bone, Calcified thyroid cartilage Cranium—full model containing nasal bone, lacrimal bone, palatine bone, ethmoid bone, vomer, zygomatic bone, inferior nasal concha	pcn.cnt.edu.pl/en/kosci-czaszki
Vertebral column	Cervical vertebrae C1-C7, Thoracic vertebrae Th1-Th12, Lumbar vertebrae L1-L5, Sacrum	pcn.cnt.edu.pl/en/kregoslup
Thoracic cage	Ribs 1–10, Sternum (Body and Manubrium)	pcn.cnt.edu.pl/en/klatka-piersiowa
Bones of upper limb	Clavicle, Scapula, Humerus, Radius, Ulna, Bones of hand (including Scaphoid, Lunate, Triquetrum, Pisiform, Trapezium, Trapezoid, Capitate, Hamate, Metacarpal bones, Phalanx bones)	pcn.cnt.edu.pl/en/kosci-konczyny-gornej
Bones of lower limb	Coxal bone, Femur, Patella, Fibula, Tibia, Bones of foot (including Talus, Calcaneus, Navicular, Medial cuneiform, Intermediate cuneiform, Lateral cuneiform, Cuboid, Metatarsal bones, Phalanx bones)	pcn.cnt.edu.pl/en/kosci-konczyny-dolnej

**Fig. 2:**
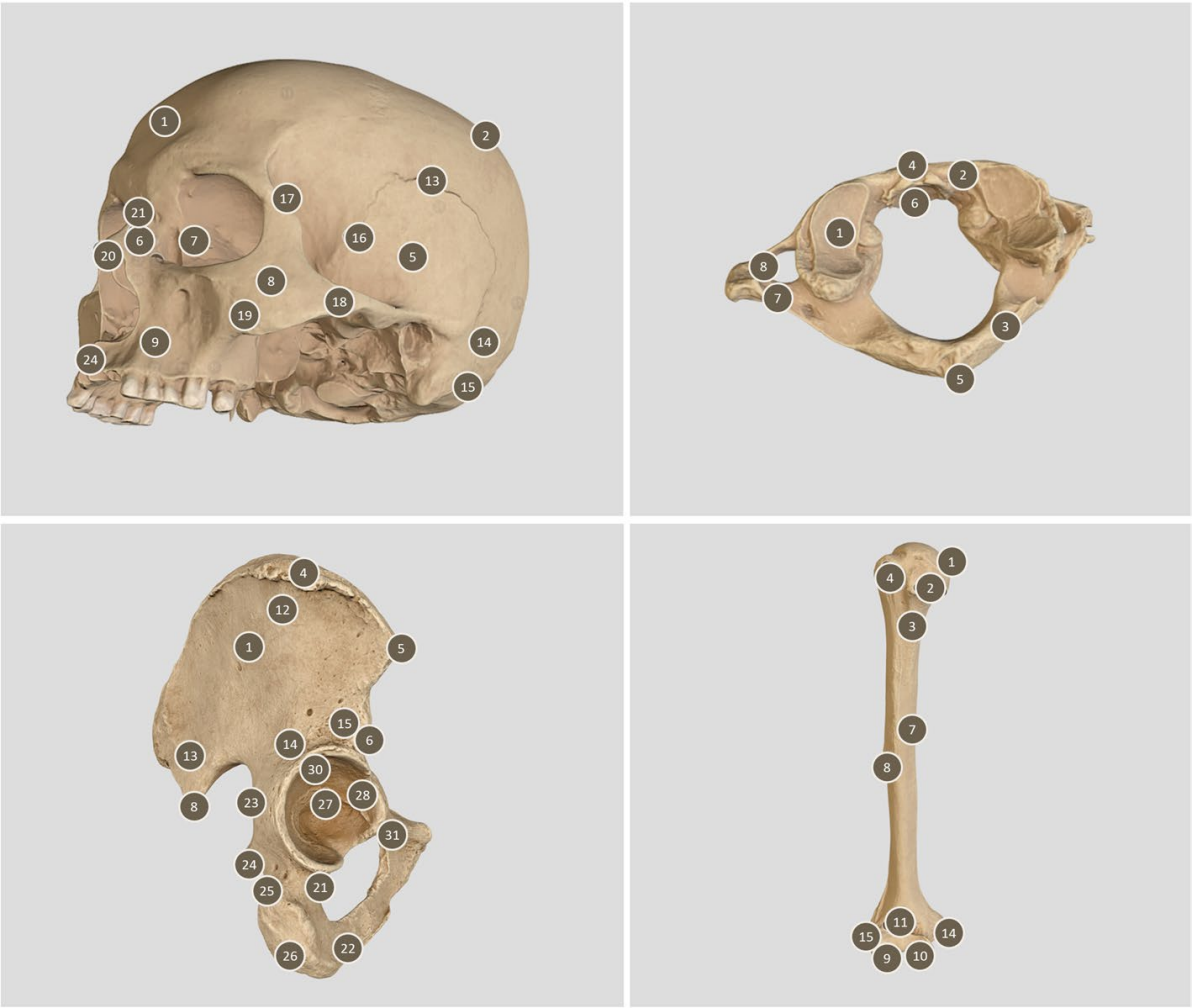
Example simplified models, available from the website, of the cranium, atlas, hip bone and humerus. This figure was prepared by the authors

Due to the integration of the API, the models are fully interactive. It is possible to rotate, scale, translate and enlarge the selected models in 3D space (Fig. 2). As a result, website implementation involves display optimization for both workstations and mobile devices. It is also possible to adapt the view to virtual or augmented resolution devices. The simplified models placed in the repository are annotated with anatomical structures according to the dictionary of anatomical names. Annotations are available in Latin, English and Polish.

**Fig. 3:**
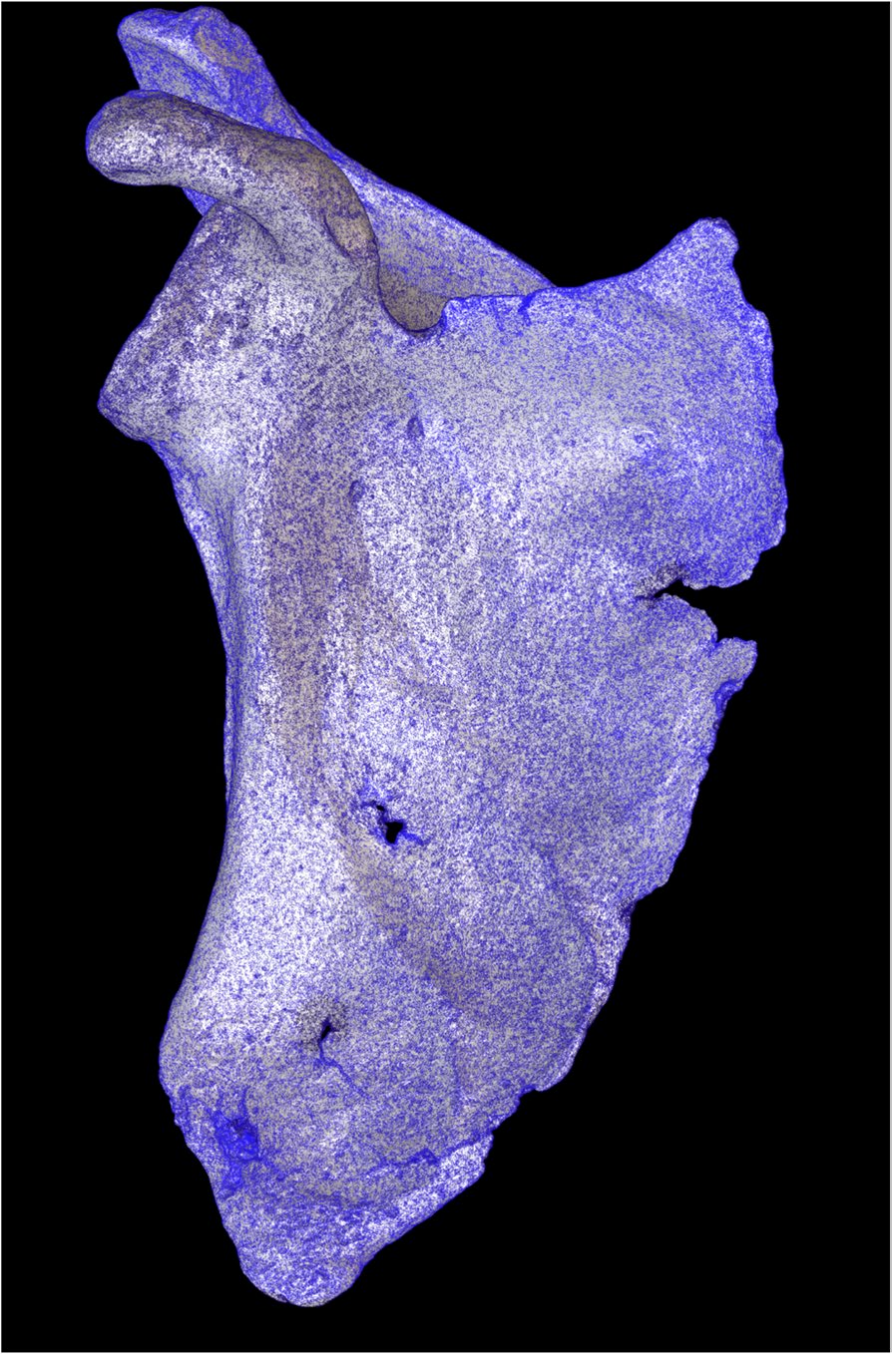
High-quality, unreduced model of the scapula with a superimposed mesh of triangles. This figure was prepared by the authors

Two hundred six full, unreduced models are stored in the database as a set of files. The geometry is stored in the Wavefront obj file format, the UV map in the mtl file format, and the texture in the jpg image format (Fig. 3). The annotations are available from the stored pac files. Access to the models is available after user registration and login. The database can be accessed, without restrictions, by any researcher wishing to use the models for noncommercial purposes. The stored models can be visualized with the open-source VisNow platform, which is also available to download from the webservice, or with any other obj viewer [15].

The database was deployed on the Ubuntu Linux distribution housing MariaDB 10.4 backend database. A virtual machine uses the Apache server (version 2.4) and personal home page (PHP) frontend (version 8.0).

#### Implications for practice

Several implications for practice for anatomy educators using the Digital Science Platform database can be considered. First, the platform facilitates conducting online classes. Students can browse models themselves, or the educator can present and analyse anatomical structures during lectures with remote conferencing tools. Second, VisNow software provides the possibility to make virtual cross-sections of the downloaded models, to create simple animations (such as rotations and zooming) and to edit the anatomical landmark annotations. These functionalities are easy to use with the VisNow Viewer 3D module.

**Fig. 4:**
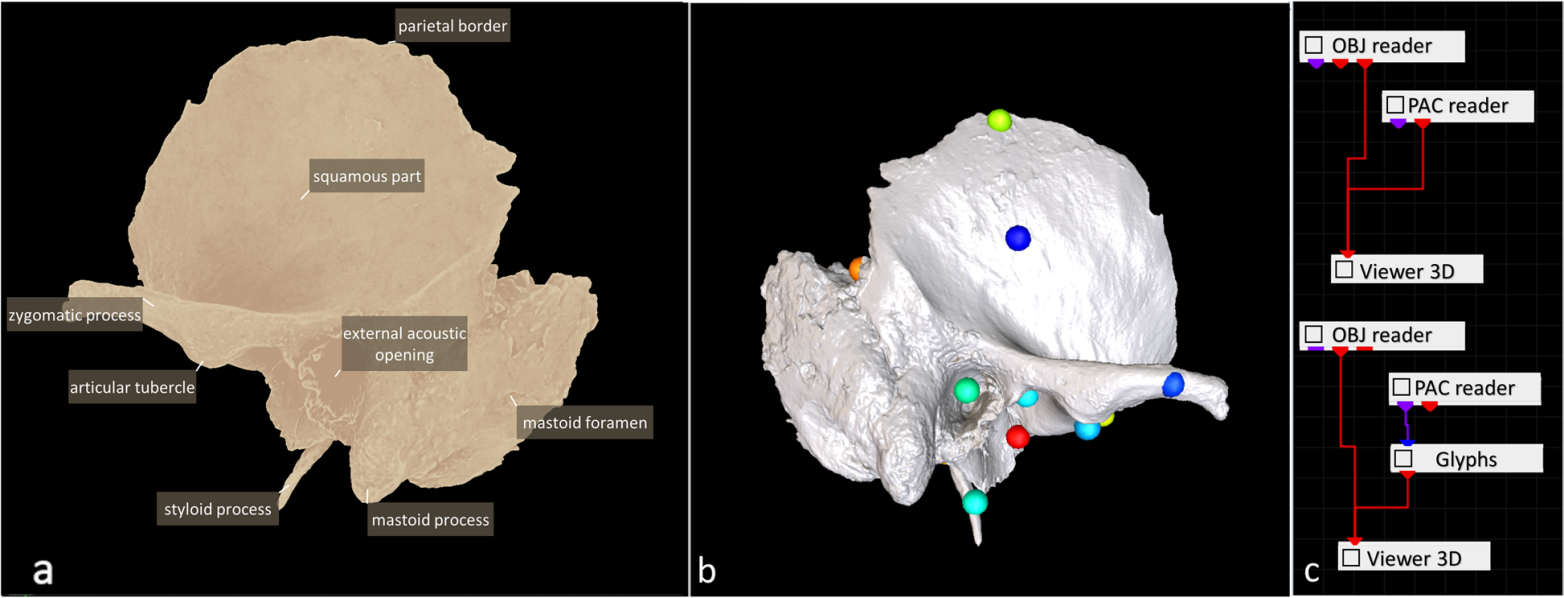
Two types of visualization high-quality models on the VisNow platform. **a** Left temporal bone with annotations, **b** right model used for revising the knowledge by the student. **c** VisNow platform interface. This figure was prepared by the authors

Furthermore, the models are based on scanned materials with some lack of bone structures due to their post-mortem destruction, which provides an opportunity for the educator to teach students in a similar way as presenting specimens available in the dissecting room. The next important element related to the educational goal of our platform involves observations resulting from the lateralization of the body. The paired structures may show slight anatomical differences resulting from, among other things, the preference to use the right or left side of the body. Therefore, the project presents separate right and left bones (Fig. 4). The models from only one site have annotations, whereas the models from the other site can be used to revise knowledge and testing. This enables students to compare and identify differences between the same bones. Such observations cannot be performed on preparations made of plastics and are unique to models from the Digital Science Platform.

The database and models were used and tested by educators and students. The greatest interest was noted during classes in anatomy, radiology and anthropology, where osteology forms the basis of further education. Academic teachers appreciated the possibility of presenting models in a three-dimensional way and the possibility of testing students' knowledge on models without marked anatomical structures.

The majority of users were students of medicine and dentistry. The database also attracted the attention of physiotherapy, nursing and archaeology students. The website has been positively received by users, which is confirmed by the number of visits of the pcn.cnt.edu.pl. It was viewed 2600 times in the last three months (from January to March 2022) by users from Poland, the United States, the United Kingdom, Germany, the Netherlands, India, and Italy. The most complex models of the cranium and pelvis were the most popular. Students found it much easier to acquire knowledge of the human skeleton thanks to the 3D visualization than with traditional atlas-based methods.

Due to the short period of time from the launch of the database, it has not been trialled; however, future evaluation of the educational utility is planned. Additionally, due to the COVID-19 pandemic and subsequent lockdowns, it was not possible to conduct scientific research using a platform. A survey of anatomy students and educators of medical universities is planned and will include an assessment of the possibility of transferring knowledge compared to traditional methods. Experimental studies will be performed among students undergoing gross anatomy practical examinations. Students will be divided into two groups: those utilizing the Digital Science Platform and those using the traditional teaching model. The groups will be compared according to the results of the examination.

The database implications for the research are related to the use of the platform in augmented and virtual reality systems. The deployment of virtual reality engines in combination with three-dimensional anatomical models from the Digital Science Platform will be the subject of research over the next few months.

### Utility and discussion

The most unique and valuable benefits of the database are related to osteological specimen access. Students of medical and paramedical faculties can use such specimens not only to learn about the structure of the human skeleton but also, above all, to create a solid foundation of medical knowledge to which they will refer during their further education and through each stage of their professional careers. Undoubtedly, the platform will be most beneficial for students attending human anatomy courses during the first years of studies in university medical faculties [16]. The interactive models will also be useful during radiology classes and clinical subjects in later years of study [8, 17]. The ability to identify anatomical structures is important when training in anthropology or bioarchaeology or for work with exhumation.

The COVID-19 outbreak has changed the perception of teaching anatomy and caused a major educational crisis due to lockdowns and the closure of medical classes [18]. To reduce the risk of the spread of the virus, the accessibility of cadavers and osteological materials was reduced. Many medical universities have reduced the acceptance of bodies during outbreaks because the donor might have been infected with COVID-19, and it was not feasible to test the cadavers [1, 2]. To avoid the risk of infection between students and teachers, most lectures and classes have been delivered online [19, 20]. Therefore, high-quality anatomical models are necessary for remote teaching [21].

It is also worth noting that the prepared bone models are not made of plastic but rather from authentic materials. This means that some fragments of the structures may have been damaged postdeposition. However, such bone fragment loss from the point of exercise is extremely important, as it enables comprehensive testing of anatomy.

The model repository is available to all research teams without restrictions. All the materials on these virtual platforms are stored with unrestricted access, featuring the ability to download each model with a web browser. Therefore, it is possible to reuse the models in research publications or educational materials.

Furthermore, the unreduced models can be viewed and visually analysed with VisNow software, which is available to download from the website after login. The full models contain up to 5 million triangles; therefore, displaying them at full resolution using the website is not possible. The VisNow software provides a rich collection of modules for loading, filtering, mapping, saving, segmenting, simulating, visualizing and processing the models. It is possible to visualize the bone structures with annotations at any resolution [22].

There are several commercially available [23–27] or free human body atlases [28–30] offering three-dimensionally reconstructed bones. The most unique feature of our database that distinguishes it from other anatomical databases is the possibility of downloading the osteological model and viewing or editing this model in other software. Other services do not allow models to be edited or reformatted [23–30]. Furthermore, osteological material from a real, scanned skeleton was used in the database; other databases are based on models that were created virtually and do not accurately represent the natural surface of the bone. The resolution of the models of other databases is comparable to the simplified models on our platform. However, to the best of our knowledge, none of the databases provides models with the resolution that is available with our unreduced models [28, 29]. Thus, it is not possible to view or print more accurate models from other services.

Further development of the Digital Science Platform will focus on adding new models of other anatomical structures (muscular, nervous, or circulatory systems). It will also include materials from rare osteological pathologies, which would be scanned in a similar manner. Furthermore, radiological models are planned to be added to the platform.

## Conclusions

An interactive web application and database of osteological material for anatomy education was developed. High-quality 3D models of anatomical structures are available to download or view on pcn.cnt.edu.pl without restrictions for all research teams and students. These database models are unique due to the specific digitalization process and skeleton specimen origin. Further development of the Digital Science Platform is foreseen in the near future to digitize other valuable materials.

## Data Availability

The datasets are available without any restrictions in the Digital Science Platform repository, pcn.cnt.edu.pl.

## Abbreviations

API: Application programming interface
PHP: Personal home page
UV map: A projection of a 2D image onto the surface of a 3D model for texture mapping
